# HIV vaccine trial safety and retention among 18-20 year olds in the HVTN 503/Phambili study support the inclusion of adolescents in future trials

**DOI:** 10.1186/1742-4690-9-S2-P131

**Published:** 2012-09-13

**Authors:** JE Volk, NA Hessol, GE Gray, JG Kublin, G Churchyard, K Mlisana, M Nchabeleng, SP Buchbinder, L Bekker

**Affiliations:** 1 University of California, San Francisco, San Francisco, CA, USA; 2 Departments of Clinical Pharmacy and of Medicine, UCSF, San Francisco, CA, USA; 3 Perinatal HIV Research Unit, University of Witwatersrand, Johannesburg, South Africa; 4 HIV Vaccine Trials Network, Fred Hutchinson Cancer Research Center, Seattle, WA, USA; 5 Aurum Institute for Health Research, Klerksdorp, South Africa; 6 Department of Medical Microbiology, University of KwaZulu-Natal, Congella, South Africa; 7 Medunsa HIV Clinical Research Unit, University of Limpopo, Limpopo, South Africa; 8 Department of Public Health, HIV Research Section, San Francisco, CA, USA; 9 The Desmond Tutu HIV Centre, University of Cape Town, Cape Town, South Africa

## Background

Worldwide, many adolescents, especially women, acquire HIV before age 18. Yet to date, no HIV vaccine trials have enrolled adolescents. Reasons for excluding adolescents from these trials include regulations protecting vulnerable subjects and concerns regarding informed consent, social harms, adverse events, and loss to follow-up.

## Methods

Using data from the HVTN 503/Phambili study, a multisite phase 2b double-blind RCT in South Africa, motivations for joining the trial, adverse events, social harms, and loss to follow-up were compared between young adults (18-20 years-old) and adults ≥21 years-old using bivariate and multivariate analyses.

## Results

Young adults (n=238) were less likely than older participants (n=563) to report joining the vaccine trial for monetary incentives (10.5% vs. 16.3%, p<0.05), and no differences were seen for altruistic motivations such as the desire to help the community (95.4% vs. 93.1%, p=.22) or to find an effective vaccine (98.3% vs. 97.0%, p=.34). There was no significant difference in social harms by age (3.8% vs. 6.9%, p=0.09). No differences were seen between 18-20 year-olds and older participants for adverse events that were definitely, probably, or possibly related to study product (5.0% vs. 7.8%, p=0.16) or for adverse events that required expedited reporting to the study sponsor (2.1% vs. 2.5%, p=0.74). Furthermore, loss to follow-up did not differ between 18-20 year-olds and older participants in either bivariate analysis (21.4% vs. 24.2%, p=.40) or in a multivariate model that controlled for both gender and study site (OR .95, CI .65-1.38).

## Conclusion

Participants who were 18-20 years old were less likely to report financial motivations for joining the HVTN 503/Phambili study and were no more likely to experience adverse events, social harms, or loss to follow-up than participants ≥21 years-old. These data are reassuring and support the safe and feasible inclusion of younger adolescents in future HIV vaccine efficacy trials.

